# Time-series transcriptome analysis mapping pulmonary immune checkpoint atlas of experimental silicosis

**DOI:** 10.1016/j.gendis.2024.101258

**Published:** 2024-03-08

**Authors:** Youliang Zhao, Yuanmeng Qi, Meixiu Duan, Changfu Hao, Wu Yao

**Affiliations:** Department of Occupational Health and Occupational Disease, College of Public Health, Zhengzhou University, Zhengzhou, Henan 450001, China

Silicosis is a chronic interstitial lung disease caused by prolonged exposure to inhalable silica particles. The initiation and progression of silicosis involve a dynamic process encompassing various cell types and molecules. Although the effector cells in different stages of silicosis undergo constant switching with disease progression, immune cells play a dominant role throughout the entire process.^1^ Therefore, comprehensively exploring cellular and molecular mechanisms underlying immune responses in silicosis becomes a prerequisite for unraveling its pathogenesis. Immune checkpoints (ICs) are crucial immunomodulatory factors that contribute significantly to the maintenance of immune tolerance and homeostasis.^2^ Our previous studies have also demonstrated that the development of experimental silicosis is accompanied by dysregulation of IC expression.^3^ Therefore, a systematic description of the dynamic expression profile of ICs in silicosis can provide a basis for subsequent experimental research. In this study, we established a mouse model of silicosis and collected lung tissue samples at various stages for transcriptome sequencing analysis. By integrating bioinformatics analysis techniques with time series-based trend analysis, we mapped out pulmonary IC atlas to highlight the critical role played by ICs in orchestrating immune responses at distinct stages of silicosis progression.

In our silicosis model, mouse lung tissues were collected after 3, 7, 14, 28, and 56 days of intratracheally instillation (4 mice per group per time point), followed by transcriptome sequencing and time series analysis. First, we screened the total differentially expressed genes (DEGs) at each observation time point (Fig. S1A, B). DEGs were screened using DESeq2 based on read count values, and the inclusion criteria for DEGs were a corrected *P*-value less than 0.05 and an absolute log2 fold change (FC) greater than 0.5.^4^ Notably, the number of DEGs was the highest at 3 days after silica exposure (6276 DEGs), while at other time points, the number of DEGs was significantly reduced (D7: 3506; D14: 2540; D28: 2732; D56: 2924). Then, we highlighted the DEGs with the most significant changes at each time point using volcano plots and heat maps (Fig. S1C–L). Describing the dynamics of gene expression over time is of great significance to fully understand gene function and regulatory mechanisms. We used the mfuzz tool to plot the time-series trend of gene expression with observation time points as abscissa and gene expression changes as ordinate, and classified them into 6 clusters (Figs. S2A–F). This visualization allowed us to clearly observe the temporal trend of DEGs, such as increase or decrease over time. Furthermore, we calculated the median value of the comparison group data in each cluster to screen out the core genes in each cluster (Fig. S2G–L). GO and KEGG enrichment analyses were performed for the genes in each cluster, and the enriched pathways included cytokine–cytokine receptor interaction, ECM-receptor interaction, oxidative phosphorylation, cell adhesion, and metabolic pathways (Fig. S3).

Secondly, the DEGs selected at each observation time point were intersected with immune-related genes (IRGs) included in the ImmPort database, and a total of 583 differentially expressed IRGs (DE-IRGs) were identified (Fig. S4A, B). The DE-IRGs at each time point were also visualized by volcano plot, and the top 10 up-regulated and down-regulated DE-IRGs obtained according to the fold change were shown by heat map (Fig. S4C–L). Next, we used ssGSEA to analyze the infiltration of immune cells in the lung tissues of mice at different time points. Among the 28 immune cell types defined, regulatory T cells, macrophages, dendritic cells, and myeloid-derived suppressor cells were significantly increased at all time points after silica exposure. On the contrary, monocytes, activated B cells, and gamma delta T cells were decreased after silica exposure (Fig. S4M). Accordingly, the temporal trends of DE-IRGs were also described and classified. The hub genes in each cluster identified by median iteration were presented by heat map (Fig. S5). GO and KEGG enrichment analyses of DE-IRGs in each cluster were also performed, and the enriched pathways mainly included cytokine–cytokine receptor interaction, Rap1 signaling, JAK-STAT signaling, TNF signaling, and NF-κB signaling (Fig. S6).

ICs are IRGs with important immunomodulatory functions and are the focus of this study. Finally, we further identified differentially expressed ICs (DE-ICs) from the selected DE-IRGs. Sixty-two genes that have been defined as ICs or their homologues were identified in the screened DE-IRGs by a literature review (Fig. 1A–F). Next, we identified DE-ICs that had the most pronounced changes in expression at each observation time point. Collectively, only *Tnfrsf9* was significantly up-regulated throughout the disease stages. In addition, *Lilr4a*, *Lilr4b*, and *Tnfsf18* were significantly up-regulated in the early and middle stages of silicosis, while *Pdcd1*, *Ctla4*, and *Tigit* were significantly up-regulated in the middle and late stages of silicosis. Furthermore, the dynamics of these identified DE-ICs were characterized and classified (Fig. 1G–N). Among them, clusters 1 and 4 had a similar trend, which was down-regulated in the early stage of silicosis and then gradually increased, while cluster 2 was just the opposite. As for cluster 3, the dynamic curve was "U-like”, with up-regulation in the early and late stages and down-regulation throughout the middle stage. The key genes in each cluster were also screened and displayed with heat maps. GO and KEGG enrichment analyses of these DE-ICs showed that the enriched pathways were mainly related to immune regulation, including cell adhesion molecules, T-cell receptor signaling, and cytokine–cytokine receptor interaction (Fig. S7).

**Figure 1:**
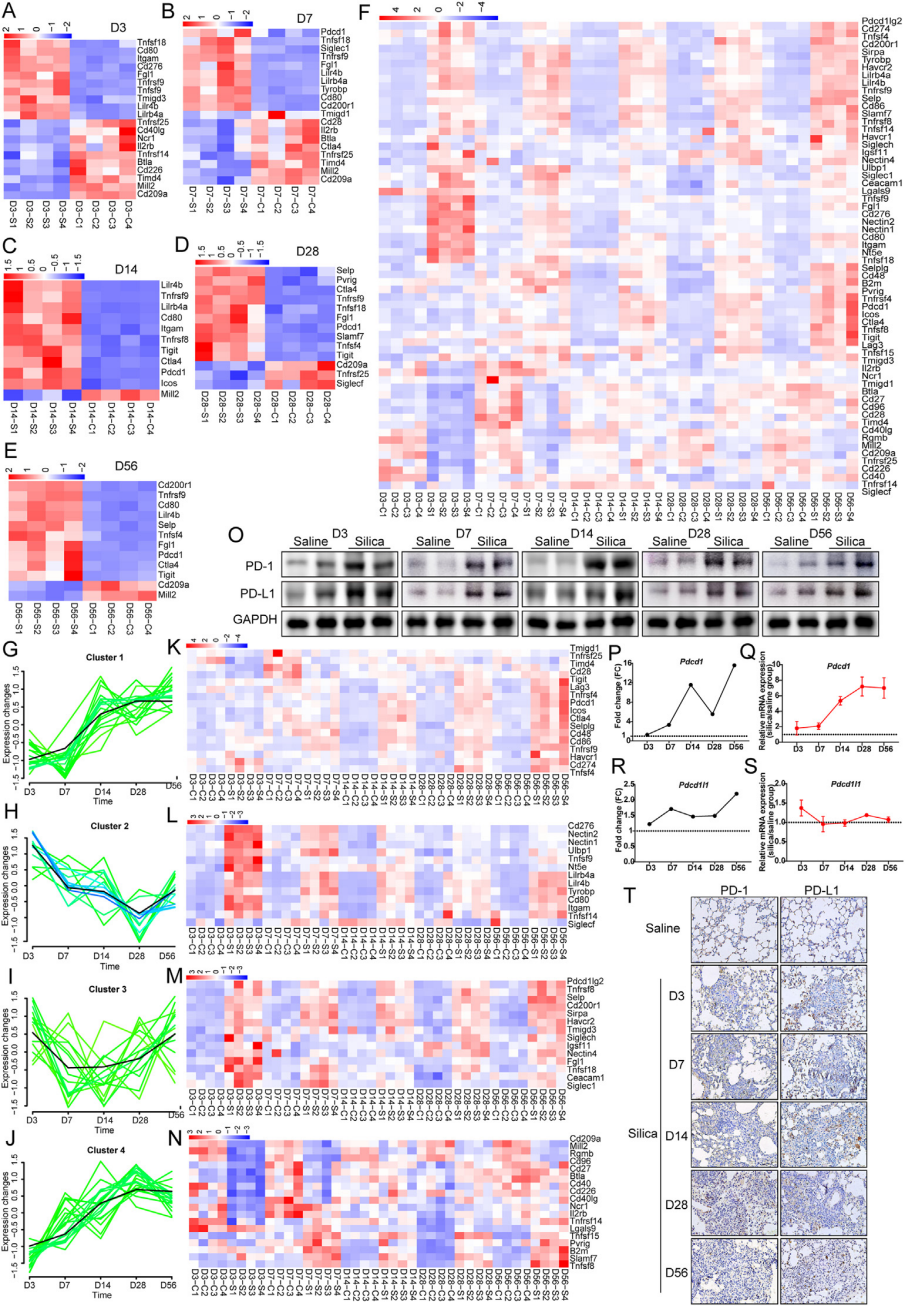
Dynamics of DE-ICs in the progression of experimental silicosis. **(A–E)** Heat map of the top 10 up- and down-regulated DE-ICs at each time point. **(F)** Heat map of total DE-ICs. **(G–J)** Clustering of DE-ICs based on time-series analysis. **(K–N)** Expression heatmap of key genes in each cluster. **(O)** Western blot analysis of PD-1 and PD-L1 in mouse lung tissues. **(P)** FC trends of *pdcd1* in sequencing data over time. **(Q)** qRT-PCR validation of *pdcd1* mRNA level. **(R)** FC trends of *cd274* in sequencing data over time. **(S)** qRT-PCR validation of *cd2*74 mRNA level. **(T)** IHC staining of PD-1 and PD-L1 in mouse lung tissues (200 × ). DE-ICs, differentially expressed immune checkpoints; FC, fold change; IHC, immunohistochemistry; PD-1, programmed cell death 1; PD-L1, programmed cell death ligand 1; qRT-PCR, quantitative real-time PCR.

Programmed cell death protein 1 (PD-1) and programmed cell death ligand 1 (PD-L1) are a pair of widely expressed ICs with important immunomodulatory functions, which have recently been confirmed to be closely related to the occurrence of a variety of fibrotic diseases.^5^ In this study, we identified that *Pdcd1* was significantly up-regulated as early as 7 days after silica exposure and showed a gradual increase trend with disease progression. For its ligands, the dynamic characteristics of *Cd274* were consistent with those of *Pdcd1*, except that its up-regulation was smaller (Fig. 1K). These results initially suggested the potential role of PD-1 and PD-L1 in the pathogenesis of silicosis, so we conducted an in-depth analysis of PD-1 and PD-L1. Firstly, the potential interacting proteins of PD-1 and PD-L1 were predicted using the STRING database, and their expression was further analyzed in correlation with PD-1 and PD-L1 (Figs. S8A–C). The expression of all the top 10 Pdcd1 interacting proteins was positively correlated with *Pdcd1*, including *Pdcd1lg2*, *Cd274*, *H2-Eb2*, *H2-Aa*, *H2-Ab1*, *Cd3g*, *Ptpn6*, *Ptpn11*, and *Lck*. However, only 5 of the top 10 Cd274 interacting proteins were positively correlated with *Cd274* expression, including *Pdcd1lg2*, *H2-Ab1*, *Cd80*, *Ptpn6*, and *Pdcd1*. Next, we used the PROMO database to predict the transcription factors that regulate PD-1 and PD-L1 transcription programs and selected the top 10 for correlation analysis (Figs. S8D–G). Transcription factors were significantly less correlated with the expression of *Pdcd1* and *Cd274* compared with interacting proteins. Notably, *Hoxa5* and *Jun* were both positively correlated with *Pdcd1* and *Cd274* expression. Finally, immunohistochemical staining, western blotting, and quantitative PCR were performed to verify the protein and mRNA expression of PD-1 and PD-L1 in the lung tissues of silicotic mice (Fig. 1O–T). The results showed that the protein expression of PD-1 and PD-L1 in the lung tissue of mice exposed to silica was significantly increased at each time point.

Taken together, this study provides dynamic observations on the characteristics of ICs during the onset and progression of silicosis through time-series transcriptomics analysis. Our findings suggest that altered expression of ICs is a rapid and widespread phenomenon in silicosis. Given the broad spectrum of IC expression, the detection of IC levels in blood cells may be helpful for early clinical diagnosis of silicosis. On the other hand, our previous study has preliminarily demonstrated that PD-1/PD-L1 inhibitors can attenuate experimental silicosis. Combined with the large number of changes in ICs observed in this study, it is suggested that ICs are expected to become a new class of targets for the clinical treatment of silicosis. In summary, due to their key immunomodulatory ability and easy detection, ICs have potential value as diagnostic markers and therapeutic targets for silicosis.

## Ethics declaration

All protocols involving animals in this study were approved by the Animal Research Ethics Committee of Zhengzhou University (No. ZZUIRB2021-123).

## Conflict of interests

The authors declare no conflict of interests.

## Funding

This work is supported by the 10.13039/501100001809National Natural Science Foundation of China (No. U1904209, 82241093).
