# Supplementary material for: Time-series transcriptome analysis mapping pulmonary immune checkpoint atlas of experimental silicosis
Source: Genes Dis. 2024 Mar 8;12(1):101258. doi: 10.1016/j.gendis.2024.101258 (PMC11532297; doi:10.1016/j.gendis.2024.101258)
Supplement: Multimedia component 1 [file mmc1.docx]

**Materials and Methods**

**Construction of experimental silicosis model**

Forty males C57BL/6 mice aged 6 ~ 8 weeks (purchased from Vital River Laboratory Animal Technology Co., Ltd, Beijing) were randomly divided into two groups. After one week of acclimatization, the mice in the silica exposure group were intratracheally instilled with 100 μL of silica suspension (diameter of silica particles was 1 ~ 5μm, 50 mg/mL), and the mice in the control group were instilled with equivalent saline. Mice were sacrificed at 3, 7, 14, 28 and 56 days after intratracheally instilled respectively. All protocols involving animals were approved by the Animal Research Ethics Committee of Zhengzhou University.

**Lung tissue collection and RNA isolation**

At each sampling time point, four mice from the control and silica groups were sacrificed and lung tissues were collected. Lung tissue homogenates were prepared using a tissue grinder, and total RNA was extracted using TRIzol reagent (Takara) according to the manufacturer's instructions. RNA concentration and purity were measured with the Nanodrop ND-2000 system (Thermo Fisher Scientific) for quality control. Only qualified RNA samples were used for the following library construction.

**Library preparation and RNA sequencing (RNA-seq)**

RNA-seq libraries were constructed with 1 μg total RNA using the ABclonal mRNA-seq Lib Prep Kit (ABclonal). Subsequently, the insert length and effective concentration of the library were analyzed by Agilent Bioanalyzer 4150 to ensure the quality of the library. Qualified libraries were sequenced on an Illumina Novaseq 6000 system and 150 bp paired-end reads were generated.

**Transcriptome data quantification and analysis**

The raw reads from sequencing facility were checked for sequencing error rate distribution and A/T/G/C content distribution to ensure data quality. The base identification error rate of all sequencing samples in this study was less than 0.05%, and the GC content was stable at about 47%. Subsequently, low quality reads with an average base quality value of less than 20 and an undeterminable number of bases greater than 5 in raw reads were filtered, and the remaining clean reads were used for subsequent analysis. The clean reads were mapped with the reference genome sequence using HISAT2 software, and the genome version used in this study was Mus_musculus_Ensembl_104(http://may2021.archive.ensembl.org/Mus_musculus/Info/Index). After excluding unmapped reads, the expression level of each gene was calculated using featureCounts software, and the results were displayed as read count and Fragments Per Kilobase of transcript sequence per Millions base pairs sequenced (FPKM) value. Next, differentially expressed genes (DEGs) were screened using DESeq2 based on read count values, and the inclusion criteria for DEGs were a corrected p-value less than 0.05 and an absolute log2 fold change (FC) greater than 0.5.

**Time-series clustering of DEGs**

After the initial screening, the DEGs at each observation time point were summarized to construct the overall differentially expressed gene set. Next, the R software package Mfuzz (the core algorithm is based on Fuzzy C-Means Clustering, version 2.60.0) was used to perform trend cluster analysis of all DEGs based on time series. The parameters for trend analysis were observation time points and FC values.

**Functional annotation and pathway enrichment of DEGs**

Gene ontology (GO) functional enrichment analysis was performed to explore the functional categories enriched by DEGs. Kyoto Encyclopedia of Genes and Genomes (KEGG) pathway enrichment analysis was performed to explore the main signaling pathways affected by DEGs. All enrichment analyses described above were performed using the R package clusterProfiler (version 4.8.2).

**Screening of differentially expressed IRGs**

The IRGs information was obtained from ImmPort database (https://www. immport. org/shared/genelists). All DEGs screened in this study were compared with IRGs in ImmPort database, and the differentially expressed IRGs (DE-IRGs) could be screened out after intersection.

**Immune cell infiltration analysis**

The gene signatures of 28 immune cells were obtained from the TISIDB database (http://cis.hku.hk/TISIDB/index.php). The R package GSVA (version 1.48.3) was used to characterize the immune infiltration of the samples in each group.

**Protein-protein interaction (PPI) network analysis**

PPI analysis was performed using the STRING database (https://www.string-db.org/). Cytoscape software (version 3.10.0, www.cytoscape.org) was used to visualize the protein interaction network.

**Transcription factor prediction and correlation analysis**

Ensembl database (https://asia.ensembl.org/) was used to obtain 2000bp sequences upstream of the promoter regions of *Pdcd1* and *Cd274* genes. Transcription factors for *Pdcd1* and *Cd274* were predicted using the PROMO database with a Maximum matrix dissimilarity rate of 1%.

**Quantitative real-time PCR (qRT-PCR)**

Quantitative PCR was performed in Quant-Studio 7 Flex Real-Time PCR system (Thermo Fisher Applied Biosystem) using TB Green® Premix Ex Taq™ II kit (Takara). The expression levels of target genes were calculated using the 2^-ΔΔCT^ method, and the results were normalized to *GAPDH.* The primer sequences used: *Pdcd1,* forward ACCCTGGTCATTCACTTGGG, reverse CATTTGCTCCCTCTGACACTG; *Cd274,* forward TGAGCAAGTGATTCAGTTTGTG, reverse CATTTCCCTTCAAAAGCTGGTC.

**Immunochemistry (IHC)**

Freshly isolated lung tissues were washed with PBS and fixed overnight in 4% paraformaldehyde followed by paraffin embedding. Then, lung tissue sections about 4 μm thickness were prepared for subsequent histological staining. For IHC staining, sections were sequentially subjected to antigen repair, endogenous peroxidase blocking, nonspecific antigen-binding site blocking, primary antibody incubation (Collagen I, 1:300; PD-1, 1:500, Servicebio; PD-L1, 1:500, Proteintech), secondary antibody incubation (horseradish peroxidase conjugated goat anti-rabbit, 1:1000), DAB coloration, hematoxylin counterstain, dehydration, clearing and mounting procedures.

**Western blotting**

Total protein was extracted from lung tissue homogenates using RIPA buffer (Dingguo), after quantification by the BCA assay ((Boster), the samples were separated by electrophoresis in polyacrylamide gels. The proteins were then transferred to PVDF membranes and blocked in 5% skim milk for 1h at room temperature. After washing, membranes were incubated in primary antibodies overnight at 4 ℃ (PD-1, 1:1000, Servicebio; PD-L1, 1:2000, Proteintech; GAPDH, 1:20000, Proteintech). Finally, membranes were incubated in HRP-conjugated secondary antibodies for 1h at room temperature. The bands were visualized using ECL chemiluminescence.

**Statistical analysis**

All statistical analyses in the present study were implemented using R software. P values were calculated by the phyper function in the R software and were considered statistically significant when they were less than 0.05.
