# Supplementary material for: Time-series transcriptome analysis mapping pulmonary immune checkpoint atlas of experimental silicosis
Source: Genes Dis. 2024 Mar 8;12(1):101258. doi: 10.1016/j.gendis.2024.101258 (PMC11532297; doi:10.1016/j.gendis.2024.101258)
Supplement: Multimedia component 2 [file mmc2.docx]

**
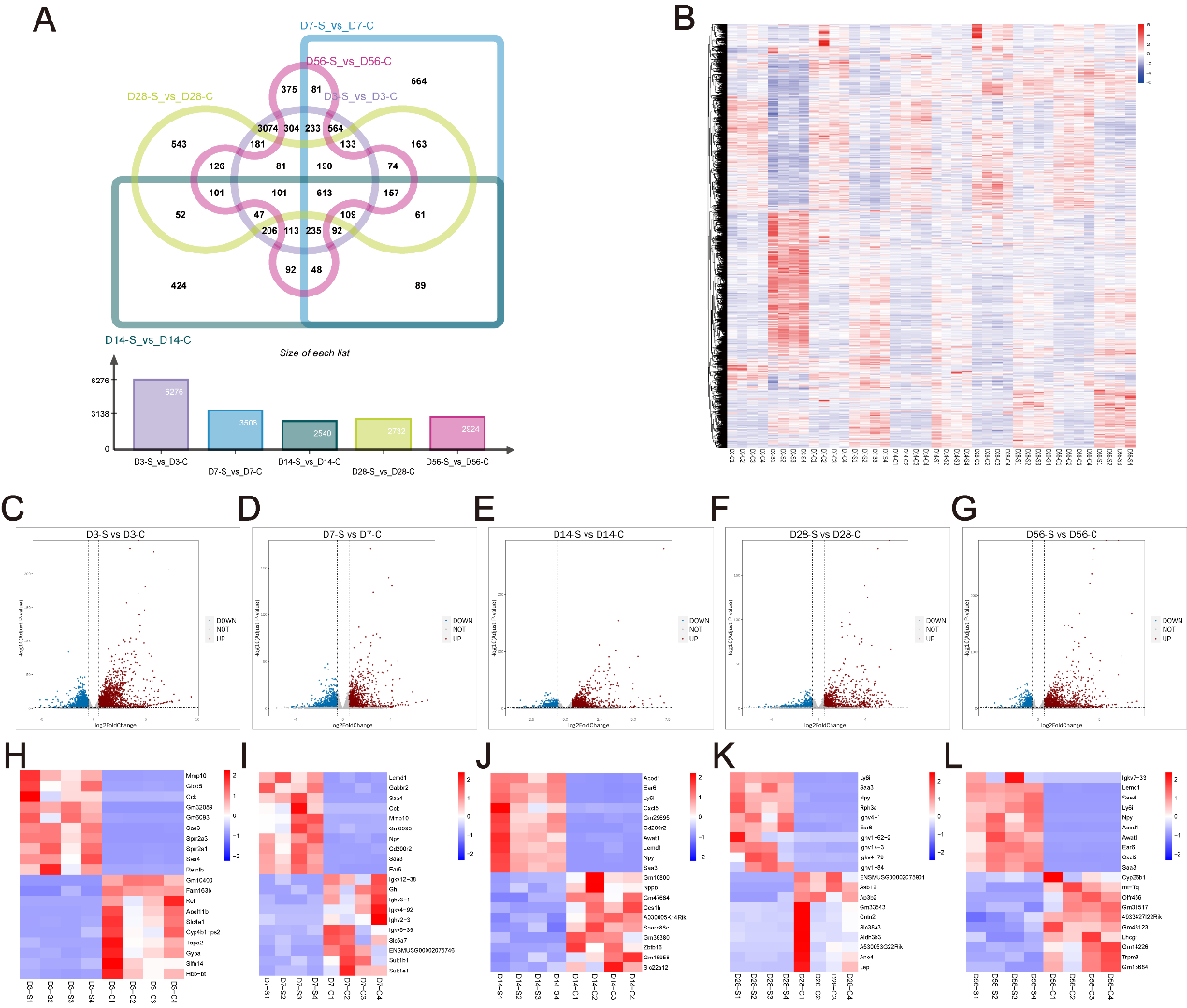
Figure. S1 Identification of DEGs at different stages of experimental silicosis.** (A) Bar and Venn plots of DEGs at each time point. (B) Heat map of all DEGs. (C - G) Volcano plot of DEGs at each time point. (H - L) Heat map of the top ten up-regulated and down-regulated DEGs at each time point.

**
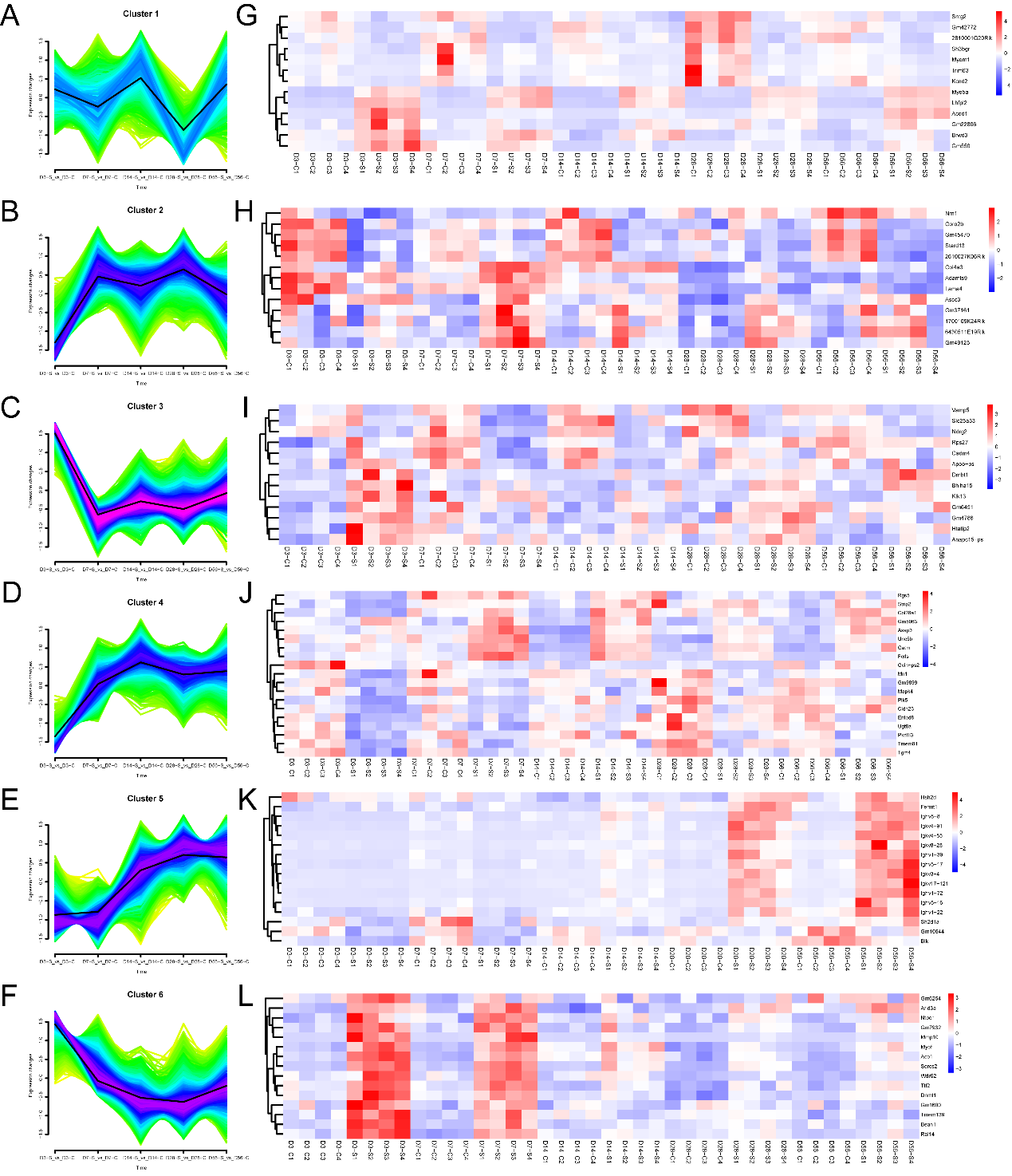
Figure. S2 Dynamics of DEGs in the progression of experimental silicosis.** (A - F) Clustering of DEGs based on time-series analysis. (G - L) Expression heatmap of key genes in each cluster.

**
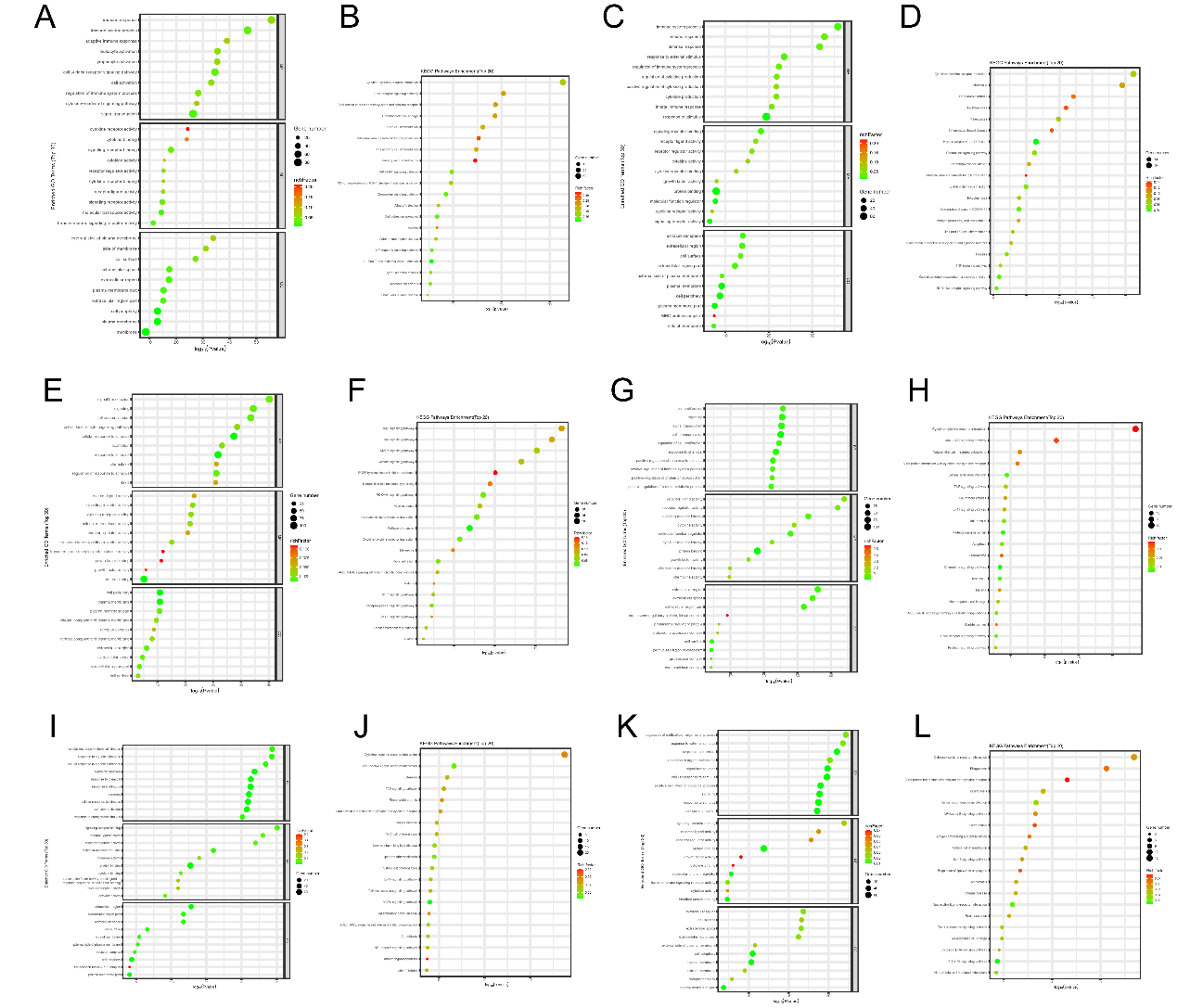
Figure. S3 GO and KEGG analysis of total DEGs in different clusters.** (A and B) Cluster1. (C and D) Cluster2. (E and F) Cluster3. (G and H) Cluster4. (I and J) Cluster5. (K and L) Cluster6.

**
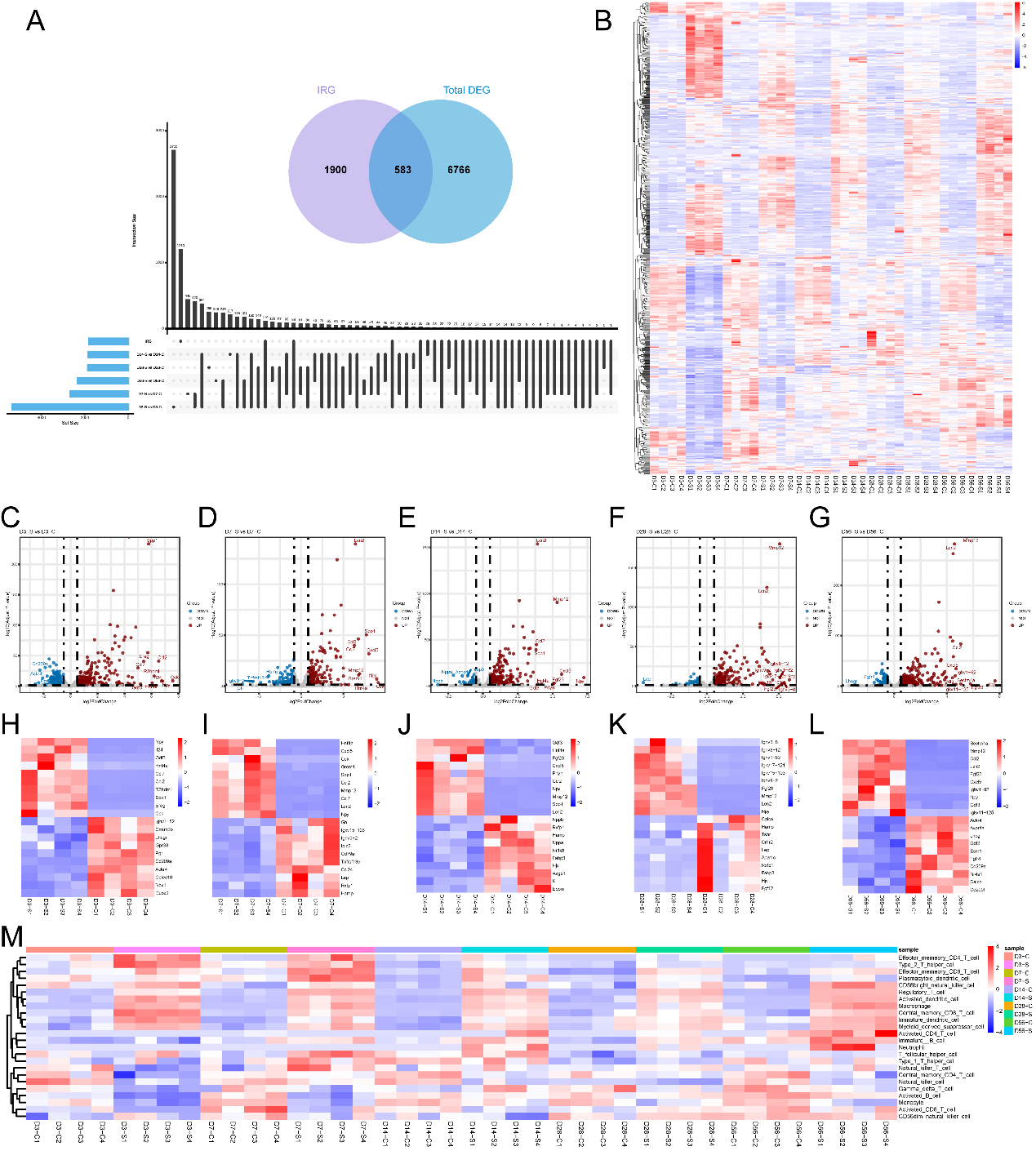
Figure. S4 Identification of DE-IRGs in experimental silicosis.** (A) Venn diagram of total DEGs and IRGs. (B) Heat map of total DE-IRGs. (C - G) Volcano plot of DE-IRGs at each time point. (H - L) Heat map of the top ten up-and down-regulated DE-IRGs at each time point. (M) Heat map of immune cell infiltration analysis.

**
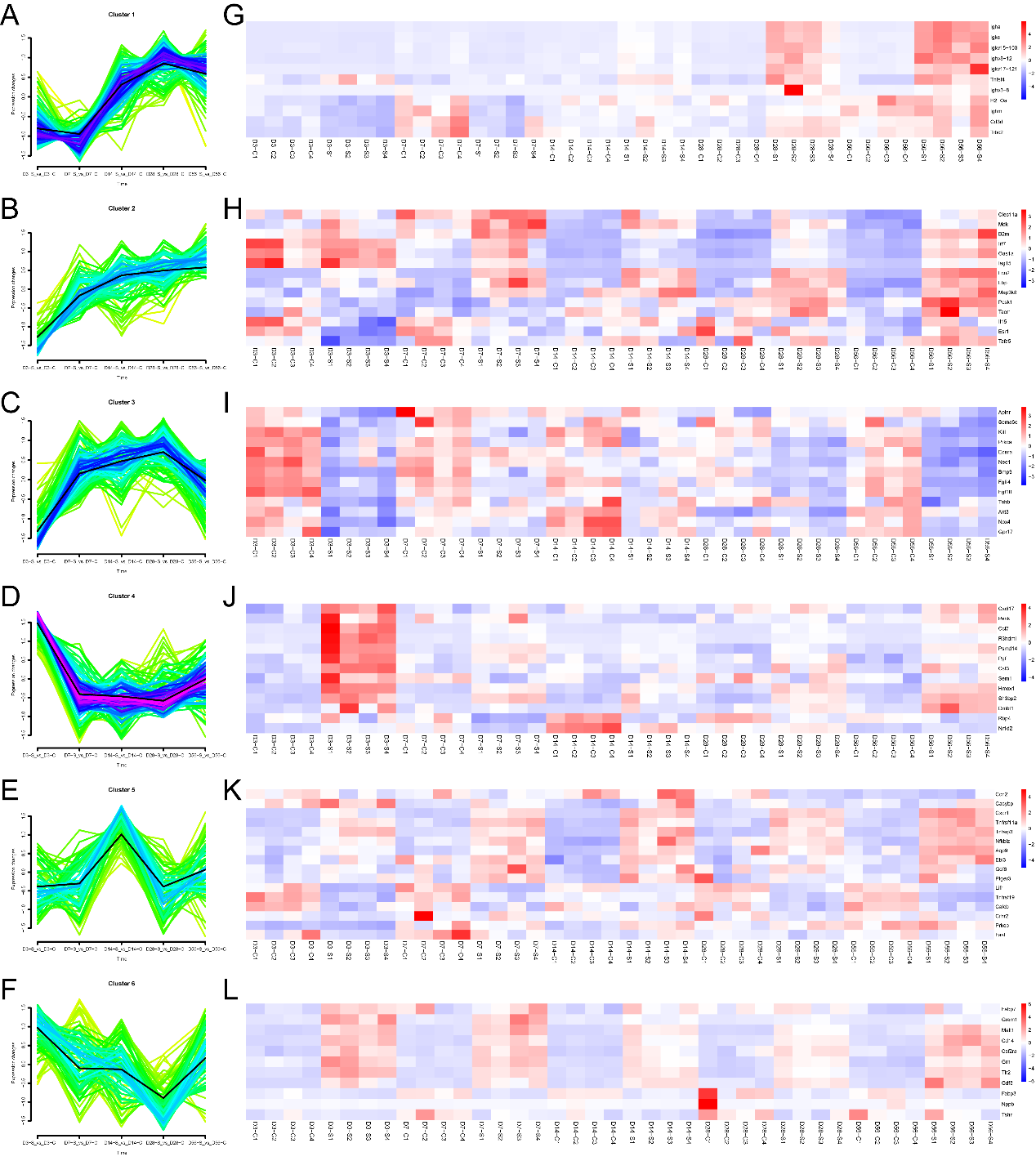
Figure. S5 Dynamics of DE-IRGs in the progression of experimental silicosis.** (A - F) Clustering of DE-IRGs based on time-series analysis. (G - L) Expression heatmap of key genes in each cluster.

**
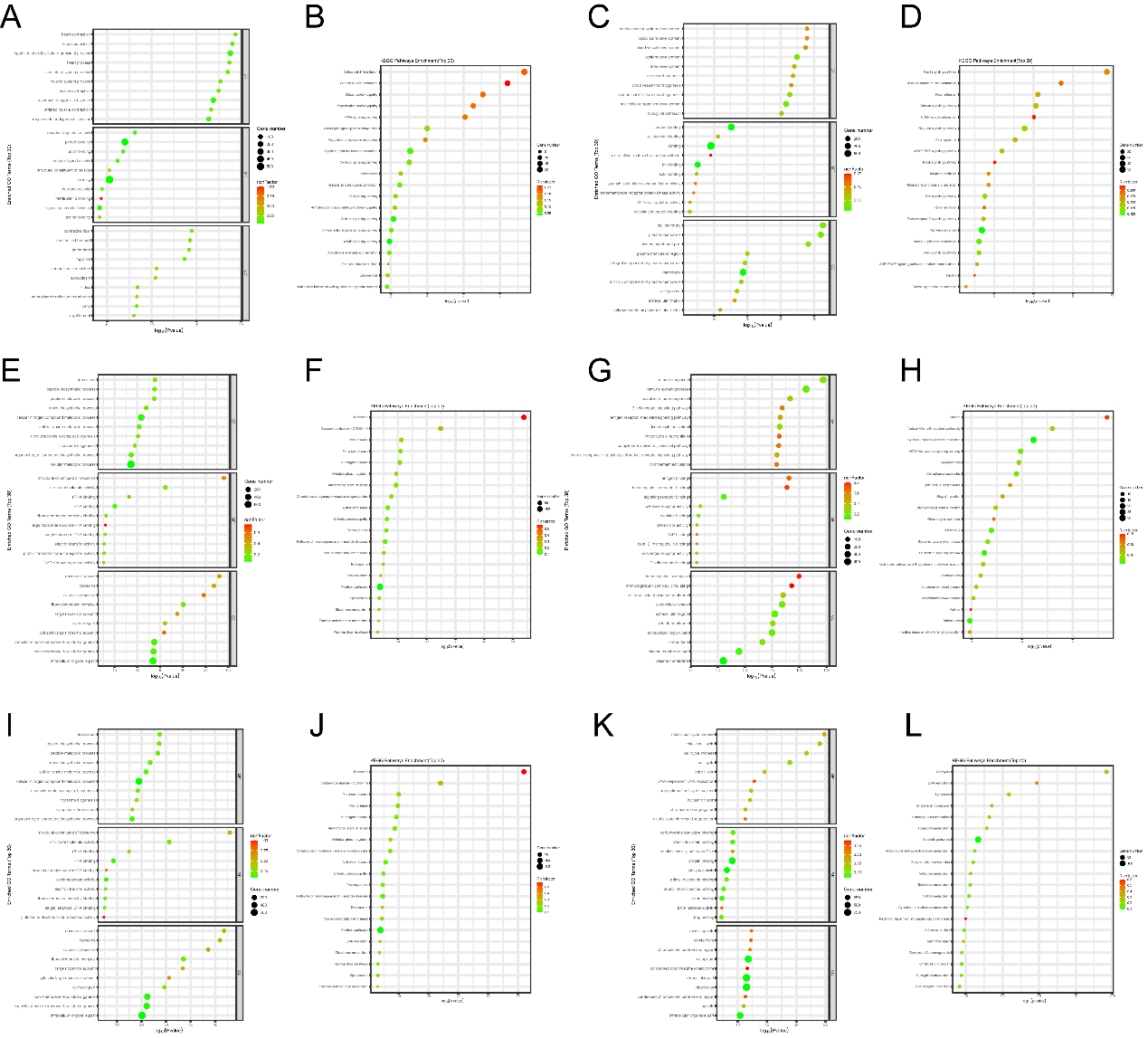
Figure. S6 GO and KEGG analysis of DE-IRGs in different clusters.** (A and B) Cluster1. (C and D) Cluster2. (E and F) Cluster3. (G and H) Cluster4. (I and J) Cluster5. (K and L) Cluster6.

**
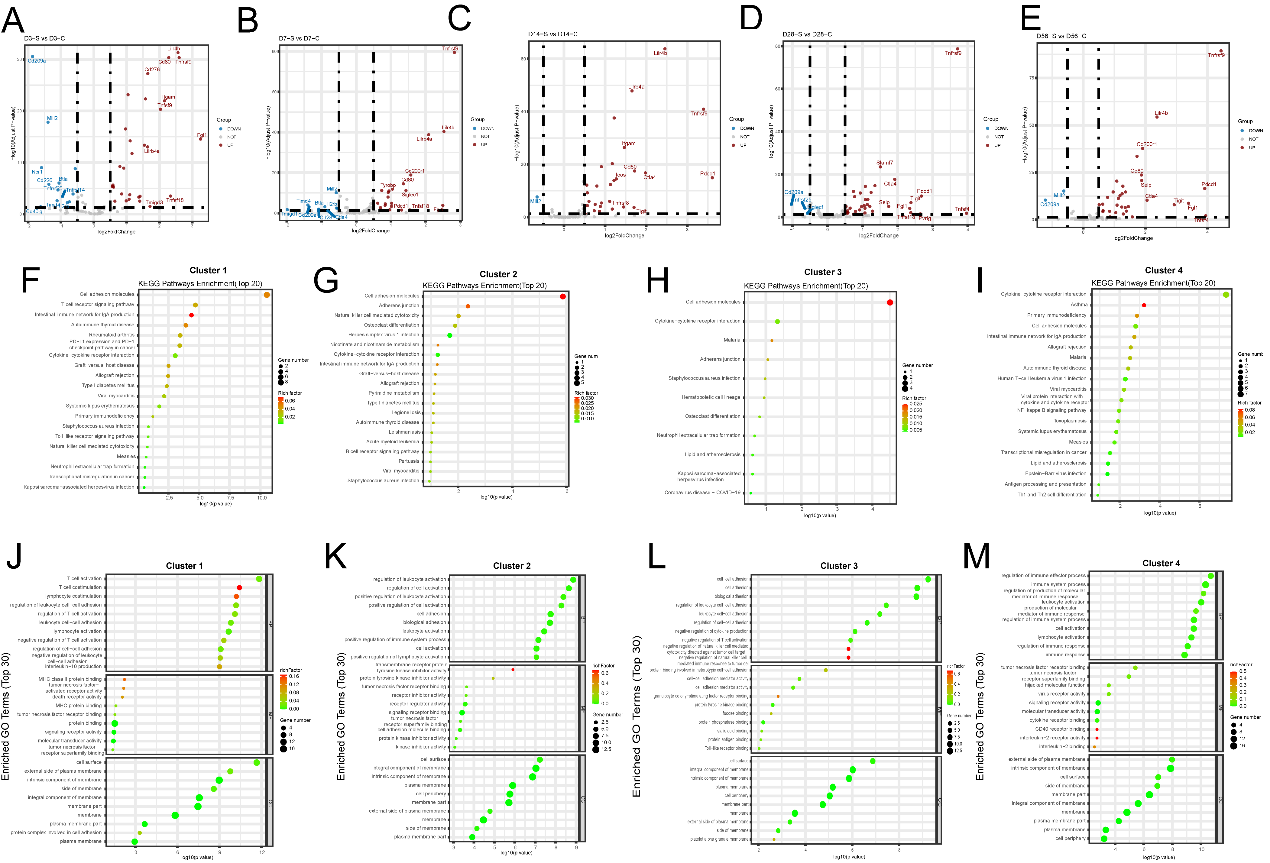
Figure. S7 GO and KEGG analysis of DE-ICs in different clusters.** (A - E) Volcano plot of DE-ICs at each time point. (F - I) KEGG pathway analysis of DE-ICs. (J and M) GO analysis of DE-ICs.


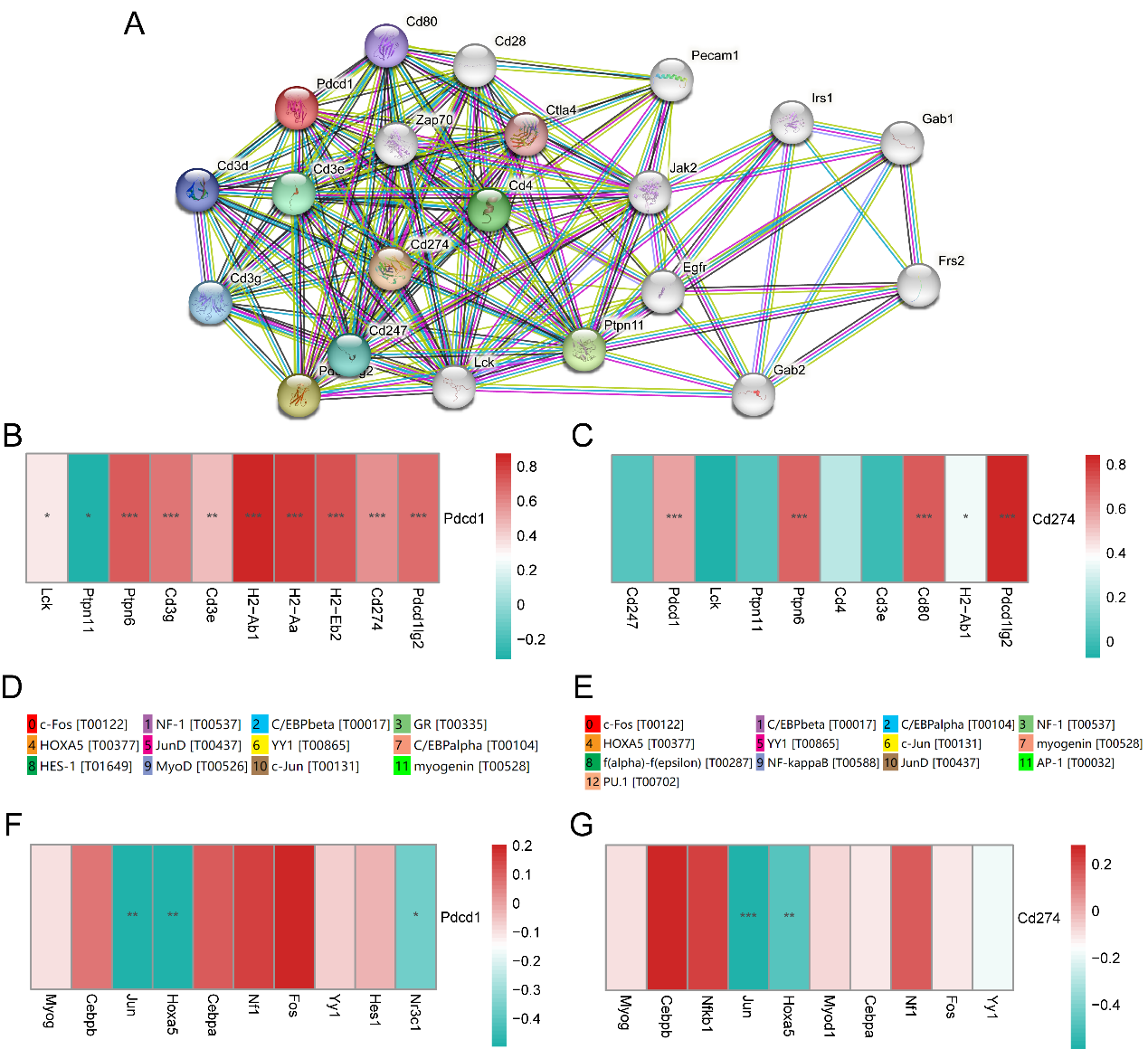
**Figure. S8 Analysis of interacting proteins and transcription factors of PD-1 and PD-L1.** (A) Interacting proteins of PD-1 and PD-L1 predicted by STRING database. (B and C) Correlation analysis of the expression of PD-1 and PD-L1 with their interaction proteins. (D and E) Key transcription factors for PD-1 and PD-L1 predicted by PROMO database. (F and G) Correlation analysis of the expression of PD-1 and PD-L1 with their transcription factors. * means statistically significant.
